# Syntax, morphosyntax, and serial recall: How language supports short-term memory

**DOI:** 10.3758/s13421-021-01203-z

**Published:** 2021-06-30

**Authors:** Judith Schweppe, Friederike Schütte, Franziska Machleb, Marie Hellfritsch

**Affiliations:** 1 University of Erfurt, Erfurt, Germany; 2 Psychology of Teaching and Learning with Digital Media, University of Passau, Dr.-Hans-Kapfinger-Str. 14b, D-74032 Passau, Germany; 3 University of Potsdam, Potsdam, Germany; 4 University of Tübingen, Tübingen, Germany

**Keywords:** Short-term memory, Working memory, Syntactic processing, Psycholinguistics, Recall

## Abstract

In the classic view of verbal short-term memory, immediate recall is achieved by maintaining phonological representations, while the influence of other linguistic information is negligible. According to language-based accounts, short-term retention of verbal material is inherently bound to language production and comprehension, thus also influenced by semantic or syntactic factors. In line with this, serial recall is better when lists are presented in a canonical word order for English rather than in a noncanonical order (e.g., when adjectives precede nouns rather than vice versa; Perham et al., 2009, *Quarterly Journal of Experimental Psychology, 62*[7], 1285–1293). However, in many languages, grammaticality is not exclusively determined by word order. In German, an adjective–noun sequence is grammatical only if the adjective is inflected in congruence with the noun’s person, number, and grammatical gender. Therefore, we investigated whether similar effects of syntactic word order occur in German. In two modified replications of Perham et al.’s study, we presented lists of three pairs of adjectives and nouns, presented in adjective–noun or in noun–adjective order. In addition, we manipulated morphosyntactic congruence between nouns and adjectives within pairs (Exp. 1: congruently inflected vs. uninflected adjectives; Exp. 2: congruently inflected vs. incongruently inflected adjectives). Both experiments show an interaction: Word order affected recall performance only when adjectives were inflected in congruence with the corresponding noun. These findings are in line with language-based models and indicate that, in a language that determines grammaticality in an interplay of syntactic and morphosyntactic factors, word order alone is not sufficient to improve verbal short-term memory.

Short-term memory refers to the temporary storage of a small amount of information to keep it available for further processing. With respect to verbal short-term memory, it is clear that it is not entirely independent from long-term linguistic representations (Baddeley, 2000; Cowan, 1999). Two opposing views of verbal short-term memory conceive it as either a dedicated store in which predominantly phonological information is maintained over the short-term, augmented by information from long-term memory (e.g., Baddeley, 2000; Page & Norris, 1998) or as a process that is parasitic on language comprehension and production (Acheson & MacDonald, 2009; D. M. Jones et al., 2006; MacDonald, 2016; Majerus, 2013; Perham et al., 2009; Saffran & Martin, 1997; Schweppe et al., 2011; Schwering & MacDonald, 2020). Both accounts are compatible with the finding that short-term serial recall of a sequence of words is much better when the words form a syntactically and semantically coherent sentence than when they do not (sentence superiority effect; Brener, 1940; see also Allen et al., 2018; Baddeley et al., 2009; Bonhage et al., 2014; Jefferies et al., 2004).

Theories that emphasize the role of a phonological store assume that this benefit is due to prelearned chunks in long-term memory. Chunks are prelearned, highly frequent sequences of items that are stored together in long-term memory, resulting in enhanced retention of material containing patterns encountered in previous experience (McNulty, 1966; Miller, 1956; Tulving & Patkau, 1962). As chunks are viewed as unitized representations, they (rather than individual items) are also assumed to underlie the capacity limits of short-term/working memory (Miller, 1956). From this perspective, sentences have a recall advantage because their components (i.e., words) can be bound into larger units. As participants have previously encountered combinations of words that constitute a syntactically and semantically coherent sentence more often than the arbitrary combinations that constitute a typical word list, more words can be remembered within the same number of chunks. Thus, syntactic and semantic information in long-term memory contribute only indirectly to short-term storage of verbal information by facilitating chunk formation.

In contrast, for language-based theories of verbal short-term memory, phonological, syntactic, and semantic information in long-term memory play a fundamental role and form the basis of short-term recall (Dell et al., 1997; D. M. Jones et al., 2006; MacDonald, 2016; Majerus, 2013; Martin et al., 1996; Potter & Lombardi, 1990; Rummer, 2003; Rummer & Engelkamp, 2001, 2011; Rummer et al., 2013; Schweppe & Rummer, 2007; Schweppe et al., 2011; Schwering & MacDonald, 2020). Upon hearing or reading a string of words, linguistic information associated with them is activated and the regular mechanisms for language comprehension and language production are applied. Schwering and MacDonald (2020), in their review of language-based models, further distinguish two types of language-based approaches that differ in the degree to which they assume that verbal working memory can be accounted for by language processing. According to “limited emergent” approaches, the language processing system provides the basis for item memory and for ordering familiar sequences, while the arbitrary ordering of letters or words in serial recall tasks is accomplished by a separate ordering component (e.g., Majerus, 2013). In contrast, “rich emergent” approaches assume that both item and order memory solely depend on language processing. For instance, MacDonald (2016) argues that utterance planning for language production provides the maintenance and ordering processes for verbal short-term memory as well. Therefore, largely automatic and well-worn routines for sentence and word assembly form the basis for recalling both sentences and unrelated word lists. However, these routines need to be overcome by means of executive control processes when the order of the memoranda mismatches that of typical sentences. While MacDonald (2016; see also Acheson & MacDonald, 2009; Schwering & MacDonald, 2020) focusses on language production, one can further argue that sentences and more sentence-like word lists benefit from language comprehension mechanisms as well. For instance, Bonhage et al. (2014) assume that, compared with arbitrary word lists, sentences allow for enriched encoding in terms of semantic and syntactic relations between items, which unburdens maintenance processes. These enriched encoding processes are similar when processing sentences for comprehension and for retention (Bonhage et al., 2017; for a similar argument see Potter & Lombardi, 1990).

These approaches not only explain the sentence superiority effect but also predict enhanced short-term memory performance below the sentence level. A study that demonstrates that even lists with rudimentary syntactic structures (and *without* semantic coherence) show a recall advantage over lists with ungrammatical sequences comes from Perham et al. (2009). They looked at serial recall of lists consisting of semantically unrelated adjectives and nouns and manipulated whether the lists were presented in a canonical word order for English—that is, an adjective preceding a noun (e.g., “insulting–roof–innocent–stomach–chubby–lily”) or in noncanonical order—that is, a noun preceding an adjective (e.g., “roof–insulting–stomach–innocent–lily–chubby”). In line with predictions from language-based accounts, Perham et al. (2009) found superior serial recall for the canonically ordered lists compared with the reversed, noncanonically ordered lists. This finding is particularly important because the difference between the list types is hard to frame in terms of chunking. Given the choice of adjectives and nouns that constitute semantically unrelated combinations (e.g., insulting roof), it is implausible to assume that the adjective–noun lists are better recalled because they benefit from prelearned chunks in long-term memory. Participants should not have encountered “insulting roof” more often than “roof insulting.” Rather, the prelearned information that is beneficial seems to be more abstract in that participants have encountered adjective–noun sequences more often than noun–adjective sequences, so that language production constraints penalize the unfamiliar and “ungrammatical” noun–adjective sequences (for a similar argument, see MacDonald, 2016). Corroborating and extending this finding, T. Jones and Farrell (2018) recently demonstrated that accuracy of serial order reconstruction for lists of adjectives, nouns, and verbs increased with conformance to English syntax when the resulting syntactic sequences were semantically meaningless.

Purely order-based manipulations of syntactic familiarity thus support language-based accounts of verbal short-term memory, both “limited emergent” ones and “rich emergent” ones. One open question is, however, whether these findings translate to languages in which a canonical order often does not suffice for word sequences to be grammatical. This is, for instance, the case in German, in which the syntactically familiar example list from above would translate to “beleidigend–Dach–unschuldig–Magen–pummelig–Lilie.” Even though adjective–noun is also the canonical word order in German, these sequences are still ungrammatical because the adjectives need to be inflected in congruence with the number and the grammatical gender of the nouns. A translation of the list in which two words each constitute a syntactically legal sequence would be “beleidigend*es*–Dach–unschuldig*er*–Magen–pummelig*e*–Lilie.” Grammaticality of the sequence thus depends on the combination of item information (i.e., morphophonological marking of the adjective) and order information (i.e., the ordering of adjective and noun).

This could also be informative regarding the distinction between rich emergent and limited emergent accounts of verbal working memory, as Schwering and MacDonald (2020) particularly emphasize the fact that different levels of language representation influence each other and are integrated. In particular, this represents a case in which item and order representations are intertwined, representing the “essential non-independence of words and word orders in utterance planning” emphasized by Schwering and MacDonald (2020, p. 8).

It is thus unclear whether, in German, a syntactically familiar word order alone is sufficient for obtaining benefits due to grammaticality or whether adjectives need to be correctly inflected for an effect of word order to occur. To address this question, we replicated Perham et al.’s (2009) experiment in German, using translations of their materials and following their procedure as strictly as possible. In addition, we manipulated the form of the adjectives: Half the participants received the lists with the adjectives in their uninflected base form (thus contrasting lists such as “beleidigend–Dach–unschuldig–Magen–pummelig–Lilie” with lists such as “Dach–beleidigend–Magen–unschuldig–Lilie–pummelig”), the other half received the same items but with correctly inflected versions of the adjectives (thus contrasting lists such as “beleidigendes–Dach–unschuldiger–Magen–pummelige–Lilie” with lists such as “Dach–beleidigendes–Magen–unschuldiger–Lilie–pummelige”).

We expect immediate serial recall of lists to be better when word pairs occur in canonical order (adjective–noun–adjective–noun–adjective–noun–adjective–noun) than when they occur in noncanonical order (noun–adjective–noun–adjective–noun–adjective–noun–adjective). This should particularly be due to a reduction in order errors. Whether such an effect is modulated by adjective inflection (uninflected vs. inflected) is an open question. While we have not preregistered predictions regarding an interaction between the manipulation of adjective–noun order and of adjective inflection, an interaction can be expected based on Schwering and MacDonald’s (2020) account: “The emergent account described here would further predict that the effect of grammatical knowledge would be moderated by semantic information of words, such as animacy, and morphophonological cues, reflecting interrelationships in LTM” (p. 9).

## Experiment 1

Experiment 1 investigated the influence of word order and adjective inflection on immediate serial recall of six-word lists by applying a 2 (adjective inflection: uninflected vs. inflected) × 2 (order: adjective–noun vs. noun–adjective) mixed design. Adjective inflection was manipulated between participants and order within participants. In addition, list version was included as a between-participants control variable: The lists that were presented in adjective–noun order in Version A were presented in noun–adjective order in Version B and vice versa. Correct serial recall (1 vs. 0) served as the binary dependent variable. In addition, we recorded the proportion of order errors and item errors. We preregistered hypotheses, methods, and analyses on AsPredicted.org (https://aspredicted.org/dm2wv.pdf).

### Method

Methods (number of participants, materials, and procedure) were chosen to resemble Perham et al.’s (2009) method as closely as possible.

#### Participants

Since we incorporated adjective inflection as an additional between factor, we doubled Perham et al.’s (2009) sample size and thus aimed to analyze 78 participants. To this end, we tested 84 native speakers of German who were compensated with course credit. Six participants were excluded from analyses due to technical problems (*n* = 4), nonnative level in German (*n* = 1), and exceeding of planned sample size (*n* = 1, last participant tested). Participants were between 18 and 39 years old with a mean age of 22.09 years (four participants did not report their age). Sixty-three participants were female, 11 male, and four participants did not report gender information.

#### Materials

The stimuli were based on Perham et al.’s (2009) materials and consisted of 24 lists with six items each in four different versions. Each list comprised three adjective–noun (or noun–adjective) pairs that were constructed such that semantic plausibility of the pairings and phonological similarity within the lists was minimized.

To maximize similarity to Perham et al.’s (2009) materials, the stimuli were translated into German. Modifications were made in some cases—for instance, when the translations led to list items being phonologically similar (same onset or rhyme) or compounds. In some cases, the inflected form of an adjective was homophonous to a noun (e.g., weite–Weite). Therefore, the directly translated adjectives were replaced with near synonyms. Further modifications were necessary because of the condition with inflected adjectives. In German, inflection of an adjective in congruence with a noun depends on the noun’s number and gender and on whether the noun phrase includes a definite article. The German language distinguishes three grammatical gender categories: masculine, feminine, and neuter. Adjectives agreeing with singular masculine nouns in an adjective–noun phrase without a definite article end with –er, with singular feminine nouns with –e, and with singular neuter nouns with –es. If a definite article is included, singular adjectives end in –e, irrespective of the gender of the noun they agree with.

It is characteristic for German that the same suffixes can illustrate different morphological functions in different contexts. For instance, the adjective ending –e can occur in both singular and plural marking of the noun, –er is also used as comparative, and all endings can appear in different cases. Therefore, some inflected word forms are more frequent than others. In general, uninflected adjectives have a higher frequency than inflected adjectives.

Furthermore, uninflected adjectives are only used in predicative or adverbial form. An example for predicative use would be “Der Clown ist lustig” [the clown is funny] and for adverbial use “Der Clown spricht lustig” [the clown talks funnily]. In contrast, inflected adjectives are used attributively as in “Er ist ein lustiger Clown” [He is a funny clown]. In a German sentence, uninflected adjectives are never followed by a noun (unless they belong to separate clauses, as in “Tee ist gesund, Kaffee nicht” [Tea is healthy, coffee is not]). Therefore, the sequence of uninflected adjective–noun order is always ungrammatical within a clause. Similarly, a noun followed by an uninflected adjective is also ungrammatical, unless the adjective is adverbial and the noun is not masculine ending on –e (“Ich finde den Kaffee lecker” [I find the coffee tasty]).

Inflection of the adjectives resulted in prolonging each adjective by one syllable. Consequently, inflection was perfectly confounded with list length, with a mean length of 1.77 syllables per item and of 10.62 syllables per list in the uninflected adjective condition and a mean length of 2.26 syllables per item and of 13.56 syllables per list in the inflected adjective condition.

To prevent participants from grouping the adjectives in the noun–adjective lists with the succeeding noun instead of the preceding one, lists were constructed such that the grammatical gender of a noun was always different from that of the preceding noun. Consequently, adjective inflection was incongruent with the succeeding noun (e.g., “Löwe_masc_–wässriger_masc_–Banane_fem_–stürmische_fem_–Mantel_masc_–besiegter_masc_”).

All of the 24 lists consisting of three adjectives and three nouns each existed in four versions: adjective–noun order with uninflected adjectives (e.g., “wässrig–Löwe–stürmisch–Banane–besiegt–Mantel”), noun–adjective order with uninflected adjectives (e.g., “Löwe–wässrig–Banane–stürmisch–Mantel–besiegt”), adjective–noun order with inflected adjectives (e.g., “wässriger–Löwe–stürmische–Banane–besiegter–Mantel”), and noun–adjective order with inflected adjectives (e.g., “Löwe–wässriger–Banane–stürmische–Mantel–besiegter”). From this, four sets of stimuli were created, two for the condition with uninflected adjectives and two for the condition with inflected adjectives. In Version A, Lists 1 to 12 were presented in noun–adjective order and Lists 13 to 24 in adjective–noun order, and in Version B, Lists 1 to 12 were presented in adjective–noun order and Lists 13 to 24 in noun–adjective order. Two additional lists were created for the practice trials, which were constructed in the same manner as the experimental lists but consisted of different words. The materials of Version A are displayed in the Appendix (Tables 1, 2, 3 and 4). The complete set of experimental lists including both versions can be found on OSF.

#### Procedure

Participants were randomly assigned to one of the four conditions (uninflected adjectives/Version A; uninflected adjectives/Version B; inflected adjectives/Version A; and inflected adjectives/Version B). They were tested individually in soundproof cubicles. The instruction and the stimuli were presented on a computer via Microsoft PowerPoint 2013. Participants were instructed to recall each list in correct serial order on a prepared response form, which contained a grid of six spaces within which to write each item. After the instruction, two practice trials were presented, one in adjective–noun order and the other in noun–adjective order. The order of the two list types and the adjective inflection (uninflected vs. inflected) varied depending on the experimental condition. After the two practice trials, the 24 experimental lists were presented. In each trial, words were presented for 700 ms. Between Items 2 and 3 and Items 4 and 5 an interstimulus interval of 1 s was introduced to emphasize association between these pairs (see also Perham et al., 2009). After presentation of each list, participants had a 10-s retention interval followed by 15 s to recall the items in the presentation order on the response forms. In the final 3 s of the retention interval and the recall phase, we additionally included a visual countdown to direct participants’ attention to the upcoming recall phase and the start of the next trial, respectively. Finally, demographic information was collected, and participants were thanked and debriefed. The experimenter was present during the instruction and the practice trials to answer any questions regarding the experimental procedure. The experiment lasted approximately 20 minutes.

#### Scoring

Participants’ written responses were digitized and scored. Responses that matched the target item in the presented list received a score of 1 and the ones mismatching a score of 0. Since the hypotheses did not include spelling specifications, scoring was case-insensitive and allowed spelling errors up to one letter unless the response was then identical to another item in the item pool or any other, correct word. A score of 0 could result from two different types of errors: When the response matched a different item in the list, the response was coded as an order error; when the response contained an item not in the respective list or the field was left empty, an item error was recorded.

#### Results

Data preprocessing, analyses and plotting for all experiments were conducted in R (R Core Team, 2020) within the environment of RStudio (RStudio Team, 2019). Analyses were primarily conducted using the package lme4 (Bates et al., 2014), while accompanying figures were mainly created with ggplot2 (Wickham, 2016).

To investigate the influence of the manipulated variables on overall recall accuracy, order errors and item errors, mixed-model logistic regression analyses were conducted, which, in contrast to a more traditional approach, with data aggregation and repeated-measures analyses of variance (ANOVAs), allow for controlling for the variance associated with random factors without data aggregation (Baayen et al., 2008). All models included order, adjective inflection, and the control factor version (A vs. B) as fixed effects (three-way interaction as well as all lower order terms). All fixed factors were coded using sum contrasts (adjective–noun order = −0.5, noun–adjective order = 0.5, finite adjective inflection = −0.5, infinite adjective inflection = 0.5, list Version A = −0.5, list Version B = 0.5). In addition to random intercepts per participant and item, all models included random slopes for order by participant and for order × adjective inflection by item. Model fit procedures in all the following analyses followed the recommendations given by Barr et al. (2013): The first model that was tried to fit was the one with the most complex random effects structure including intercepts and slopes, which reduces the probability for Type I errors. If this model failed to converge, the random effects structure was successively reduced until the model converged. The first model that converged was taken as the best fit. The analysis code can be accessed via the OSF project (https://osf.io/r6x4v/).

To parallel Perham et al.’s (2009) analyses, we additionally conducted an ANOVA including the within-subject factors order (adjective–noun vs. noun–adjective) and serial position (1 to 6) and the between-subject factor list version (A vs. B) plus our additional between-subject factor adjective inflection (uninflected vs. inflected). Since there was a strong overlap of results and since the aggregated data were not normally distributed, we refrain from reporting the ANOVA results in the main text. Instead, the code and results can be found on the OSF repository.

We first report data for our main dependent variable, overall recall accuracy. In addition, we conducted separate analyses for order and item errors.

#### Overall recall accuracy

**Fig. 1:**
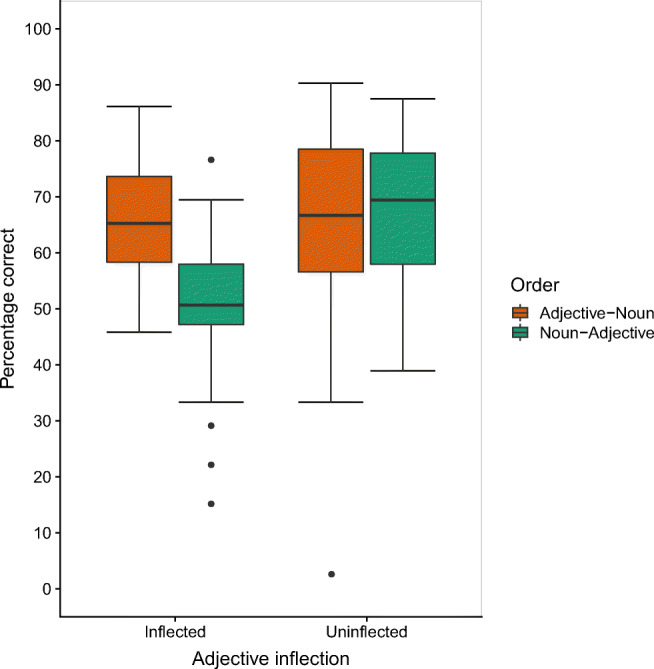
Percentage of items recalled correctly in Experiment 1 as a function of order and adjective inflection. The standard boxplot with whiskers with maximum 1.5 IQR is based on observed data.

Overall accuracy was defined as the accuracy of recalling an item in its original position. On average, participants recalled 63% (*SD* = 13) of the items across all conditions. Figure 1 shows the percentage of items recalled per adjective inflection × order group (for an additional serial position plot, see Fig. 3 in the Appendix).

As fixed effects, the model included order, adjective inflection, and version (main effects and interaction). There were significant main effects for order, β = −0.37199, *SE* = 0.07557, *z* = −4.922, *p* < .001, and adjective inflection, β = 0.44493, *SE* = 0.15429, *z* = 2.884*, p* = .004, while the control variable version did not significantly affect overall recall accuracy, β = −0.06319, *SE* = 0.14993, *z* = −0.421*, p* = .673. Serial recall of lists in adjective–noun order (66%, *SD* = 14) was superior to that of lists in noun–adjective order (59%, *SD* = 15), and accuracy was higher for lists with uninflected adjectives (67%, *SD* = 14) than for lists with inflected adjectives (58%, *SD* = 10).

However, these main effects were qualified by an interaction between order and adjective inflection (β = 0.84806, *SE* = 0.15330, *z* = 5.532, *p* < .001). An advantage for adjective–noun order over noun–adjective order was observable only in the condition with inflected adjectives (66%, *SD* = 11 vs. 51%, *SD* = 13; β = 0.7960, *SE* = 0.106, *z* = 7.482, *p* < .001), while there was no order difference when adjectives were uninflected (adjective–noun: 66%, *SD* = 17, vs. noun–adjective: 68%, *SD* = 12; β = −0.0520, *SE* = 0.109, *z* = −0.478, *p* = .964). Surprisingly, this interaction did not reflect an advantage for the most grammatical condition with adjective–noun order and inflected adjectives compared with all other conditions, but rather a disadvantage for the noun–adjective condition with inflected adjectives compared with all other conditions. A likelihood ratio test confirmed that the model including this interaction was significantly better than one without it, χ^2^(2) = 25.977, *p* < .001.^1^ None of the remaining interaction terms was significant (all *p*s > .391).

#### Order errors

Order errors were defined as items that were recalled correctly but in an incorrect position. The overall proportion of order errors was at 4% (*SD* = 3).

The logistic mixed-effects model concerning order errors had the same fixed effects structure as the overall accuracy model. There was a significant main effect for order, β = 0.7640, *SE* = 0.2259, *z* = 3.381, *p* < .001, while neither adjective inflection (β = 0.3594, *SE* = 0.2244, *z* = 1.602*, p* = .109), nor the control variable version (β = 0.2069, *SE* = 0.2092, *z* = 0.989*, p* = .323) significantly affected order errors. There were fewer order errors in lists with adjective–noun order (3%, *SD* = 4) than in lists with noun–adjective order (5%, *SD* = 5). Of the interactions, only the two-way interaction between order and version reached significance, β = −1.4165, *SE* = 0.4493, *z* = −3.153*, p* = .002 (all remaining interactions: *p*s > .238). Participants made fewer order errors in the adjective–noun lists (2%, *SD* = 3) compared with the noun–adjective lists (6%, *SD* = 6) only in Version A, in which they began with the noun–adjective lists (β = −1.4722, *SE* = 0.338, *z* = −4.361, *p* < .001). In Version B, in which the order of the blocks was reversed, there was no significant difference (noun–adjective: 3%, *SD* = 3 vs. adjective–noun: 4%, *SD* = 4; β = −0.0557, *SE* = 0.298, *z* = −0.187, *p* = .998). A likelihood ratio test confirmed that the model including this interaction between order and list version was significantly better than one without it, χ^2^(2) = 9.9313, p = .007.^2^

Given that our manipulations combined an order-based manipulation and an item-based one, we defined order errors irrespective of the number of items recalled. However, the high proportion of item errors and the differences between conditions in the proportion of item errors (see further below) may have impacted our measure. Therefore, we additionally report conditional probabilities for order errors in each of the four conditions of interest (order × inflection) by dividing the total number of order errors in a list by the total number of items recalled for that list, independent of their order (Murdock, 1976; Poirier & Saint-Aubin, 1996; Saint-Aubin & Poirier, 1999).^3^ The pattern of conditional probabilities was very similar to our main descriptive summaries of absolute numbers of order errors above. The conditional probability for an order error occurring in the adjective–noun conditions (inflected: .054; uninflected: .061) was slightly smaller than in the noun–adjective conditions (inflected: .10; uninflected: .08), but there were no descriptive differences between inflection conditions. Moreover, even conditional probabilities for order errors were rather low overall.

#### Item errors

Item errors comprised all occurrences in which participants either did not respond (omission error), responded with a word that was not in the list (extra-list intrusion), or in which they modified a word in the list (e.g., inflection errors). The overall proportion of item errors was at 34% (*SD* = 12).

The logistic mixed-effects model concerning item errors had the same fixed effects structure as the overall accuracy. It revealed significant main effects for order, β = 0.29800, *SE* = .06592, *z* = 4.520, *p* < .001, and adjective inflection, β = −0.48752, *SE* = 0.14195, *z* = 3.434, *p* < .001. The main effect for the control variable version did not reach significance (β = 0.06865, *SE* = 0.13601, *z* = 0.505, *p* = .614). There were fewer item errors in lists with adjective–noun order (31%, *SD* = 13) than in lists with noun–adjective order (36%, *SD* = 13). In addition, participants committed fewer item errors in lists with uninflected adjectives (29%, *SD* = 12) than in lists with inflected adjectives (39%, *SD* = 8). The main effects for order and adjective inflection were, however, qualified by a significant interaction between the two factors, β = −0.79664, *SE* = 0.14221, *z* = −5.602, *p* < .001. The advantage for adjective–noun lists over noun–adjective lists was observable only when the adjectives were inflected (32%, *SD* = 9, vs. 45%, *SD* = 10; β = −0.6963, *SE* = 0.0945, *z* = −7.369, *p* < .001), while this was not the case in lists with uninflected adjectives (β = 0.1003, *SD* = 0.0994, *z* = 1.010, *p* = .744). Descriptively, there was even an advantage for noun–adjective lists (30%, *SD* = 16) over adjective–noun lists (28%, *SD* = 11) when adjectives were uninflected. Just like in the model for overall accuracy, the interaction between order and adjective inflection did not reflect an advantage for the most grammatical condition with adjective–noun order and inflected adjectives compared with all other conditions, but rather a disadvantage for the noun–adjective condition with inflected adjectives compared with all other conditions. A likelihood ratio test confirmed that the model including this interaction was significantly better than one without it, χ^2^(2) = 26.756, *p* < .001.^4^ None of the remaining interaction terms reached significance (all *p*s > .518).

### Discussion

We replicated Perham et al. 's (2009) experiment in German, additionally manipulating whether the adjectives were presented in uninflected form or inflected in congruence with the corresponding noun’s grammatical gender and number. Like Perham et al. (2009), we found an advantage for lists presented in canonical (i.e., adjective–noun) order, but only when the adjective–noun sequences were congruent and therefore grammatically correct. Word order alone was thus not sufficient to improve serial recall when the two-word sequences were otherwise ungrammatical. In Perham et al.’s (2009) experiment, there was no such difference, because in English the adjectives did not need to be inflected to form a syntactically correct sequence with the nouns in their base forms. Our findings indicate that syntactic constraints on word order that can benefit serial recall of word lists are intertwined with other (morpho-) syntactic constraints such as adjective inflection.

Even though the interaction between word order and adjective inflection is in line with language-based models of short-term memory, the exact data pattern is surprising. If the combination of a canonical word order and correctly inflected adjectives makes it easier to recall the items in their correct serial order, performance in this condition should have been better than in all other conditions. However, the only condition that differed was the one with noncanonically ordered items and inflected adjectives. In other words, rather than an advantage for canonical word order that was restricted to lists with inflected adjectives, we found a disadvantage for noncanonical word order that was restricted to lists with inflected adjectives. How can this unexpected pattern be accounted for? One reason may be a confound in our experiment that is due to morphosyntactic constraints of the German language: the inflected adjectives were longer than their uninflected base forms. Depending on grammatical gender, typically between one and three phonemes/letters need to be added and this goes along with the addition of another syllable (e.g., wässrig vs. wässriger_masc;_ see also Materials). In addition, inflected forms are less frequent than uninflected forms. Thus, there might be an overall disadvantage for the lists with inflected adjectives in terms of a word-length effect and a frequency effect. These potential word length and frequency effects may then have masked an advantage for the most grammatical condition with adjective–noun lists and inflected adjectives. Moreover, one could argue that an overall advantage for the uninflected condition was due to participants having more information to track than in the uninflected condition, as both the adjectives’ stems and the specific inflections had to be remembered.

An opportunity to compare morphosyntactically correct and incorrect adjective–noun phrases while controlling for word length is to use incongruently inflected adjectives. Therefore, we conducted a modified replication of Experiment 1, in which we contrasted congruently and incongruently inflected adjectives to test this post hoc assumption. Like in Experiment 1, the adjective–noun phrases were grammatically incorrect despite the canonical order, and unlike Experiment 1, syllable-based word length was the same in the two adjective inflection conditions.

Based on the results of Experiment 1 and the account put forward by Schwering and MacDonald (2020), we expect an interaction between order and adjective inflection in immediate serial recall of word lists comprised of noun–adjective pairs. In particular, recall of lists comprised of three syntactically familiar adjective–noun pairs with congruent adjective inflection should be superior to recall of lists in adjective–noun order with incongruently inflected adjectives and to recall of lists in noun–adjective order.

## Experiment 2

Experiment 2 was based on a 2 (adjective inflection: congruently inflected vs. incongruently inflected) × 2 (order: adjective–noun vs. noun–adjective) mixed design. Adjective inflection was manipulated between participants and order within participant. In addition, list version was included as a between-participants control variable: The lists that were presented in adjective–noun order in Version A were presented in noun–adjective order in Version B and vice versa. Correct serial recall served as the dependent variable. In addition, we recorded the proportion of order errors and item errors. We preregistered hypotheses, methods, and analyses on AsPredicted.org (https://aspredicted.org/ud5j2.pdf).

### Method

Methods (number of participants, materials, and procedure) were chosen to resemble Perham et al.’s (2009) method and Experiment 1 as closely as possible.

#### Participants

A power simulation (simR package Green & MacLeod, 2016) with the data from Experiment 1 revealed that to find the interaction effect of interest (effect size of .5 with 80% power, with confidence intervals above the 80% level), we needed at least 70 participants. Thus, 77 participants were tested and seven participants then excluded from analyses due to nonnative level in German (*n* = 2) and exceeding of planned sample size (*n* = 5, last participants tested). Participants were between 18 and 35 years old with a mean age of 22.6 years. Sixty-two participants were female.

#### Materials

The stimuli were based on the ones used in Experiment 1, but instead of uninflected adjectives and congruently inflected adjectives, we contrasted incongruently and congruently inflected adjectives. This modification required a few changes in the stimuli. As in Experiment 1, adjective inflection was incongruent with the nouns from adjacent pairs. In the condition with incongruently inflected adjectives, this implied that adjectives had to be incongruent with both the noun from the adjective–noun or noun–adjective pair and the adjacent pair. However, the identity of the adjacent noun differed depending on the order condition: The adjectives were adjacent to the noun from the preceding pair in the adjective–noun condition, but adjacent to the noun from the subsequent pair in the noun–adjective condition. This could only be achieved by having one or two of the adjectives in each list occurring in different forms in the adjective–noun condition and in the noun–adjective condition (e.g., “wässrige_[fem]_*–*Löwe_[masc]_–stürmisches_[neut]_–Banane_[fem]_–besiegtes_[neut]_*–*Mantel_[masc]_” vs. “Löwe_[masc]_–wässriges_[neut]_–Banane_[fem]_–stürmisches_[neut]_–Mantel_[masc]_– besiegte_[fem]_”). In addition, some nouns used in Experiment 1 had to be substituted because of their grammatical gender or because they could be interpreted as both singular and plural. As adjective inflection in German is the same for feminine singular nouns and for plural nouns of any grammatical gender (“−e”), we substituted those nouns in all conditions to avoid apparent plural congruence with feminine adjectives. Since in German the use of adjective endings depends on number and case, and endings can be ambiguous, certain inflected word forms are more frequent than others (see Materials, Experiment 1). Therefore, the gender distribution was similar between the different order conditions in Experiment 2 (adjective–noun: 17 masc, 9 neut, 10 fem; noun–adjective: 17 masc, 8 neut, 11 fem).

All of the 24 lists existed in four versions: adjective–noun order with congruently inflected adjectives (e.g., “wässriger–Löwe–stürmische–Banane–besiegter–Mantel”), noun–adjective order with congruently inflected adjectives (e.g., “Löwe–wässriger–Banane–stürmische–Mantel–besiegter”), adjective–noun order with incongruently inflected adjectives (e.g., “wässrige–Löwe–stürmisches–Banane–besiegtes–Mantel”), and noun–adjective order with incongruently inflected adjectives (e.g., “Löwe–wässriges–Banane–stürmisches–Mantel–besiegte”). From this, four sets of stimuli were created, two for the condition with congruently inflected adjectives and two for the condition with incongruently inflected adjectives. As in Experiment 1, Lists 1 to 12 were presented in noun–adjective order and Lists 13 to 24 in adjective–noun order in Version A, and in Version B, Lists 1 to 12 were presented in adjective–noun order and Lists 13 to 24 in noun–adjective order. Two additional lists were constructed for the practice trials, which were constructed in the same manner as the experimental lists but consisted of different words. The materials of Version A are displayed in the Appendix (Table 5, 6, 7 and 8). The complete set of experimental lists including both versions can be found on OSF.

#### Procedure

The procedure matched that of Experiment 1, except for the following: The instruction and the stimuli were presented via Inquisit, and recall was typed instead of handwritten. Specifically, following the presentation of each list and the 10-s retention interval, the word “Wiedergabe” (recall) appeared on the upper left side of the screen. Beneath, six textboxes labeled with each of the positions were provided, and participants were instructed to reproduce the items in the correct order. We prolonged the recall phase to 20 s because of the longer mean list length. Instead of the visual countdown, the beginning of the recall phase was announced via a ring-out.

#### Scoring

Participants’ written responses were scored analogously to Experiment 1.

### Results

Again, mixed-model logistic regression analyses were conducted, using both participant and item as random effects and sum coding for all fixed factors. The code (which can be accessed via the OSF project) gives information about the specific steps to convergence and random effects structure for each model. Likewise, the results of an ANOVA can be found in the same repository. We first report data for our main dependent variable, overall recall accuracy. In addition, we conducted separate analyses for order and item errors.

#### Overall recall accuracy

**Fig. 2:**
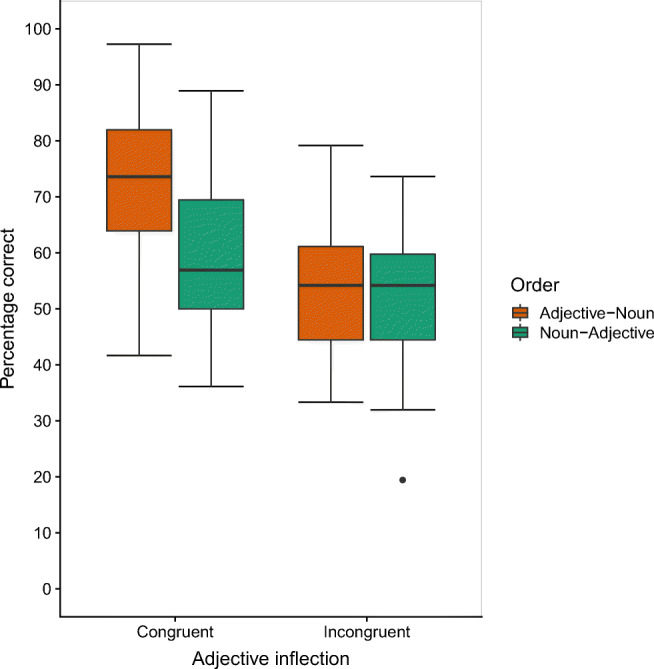
Percentage of items recalled correctly in Experiment 2 as a function of order and adjective inflection. The standard boxplot with whiskers with maximum 1.5 IQR is based on observed data.

**Fig. 3:**
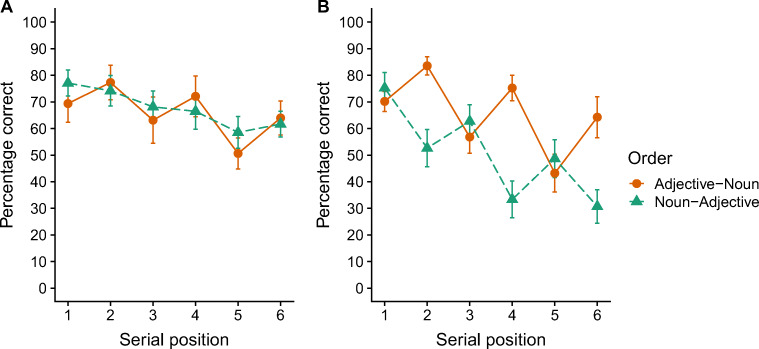
Overall accuracy in Experiment 1 as a function of serial position and order with uninflected adjectives (**a**) and inflected adjectives (**b**). Figures are based on observed data and error bars indicate 95% confidence intervals.

**Fig. 4:**
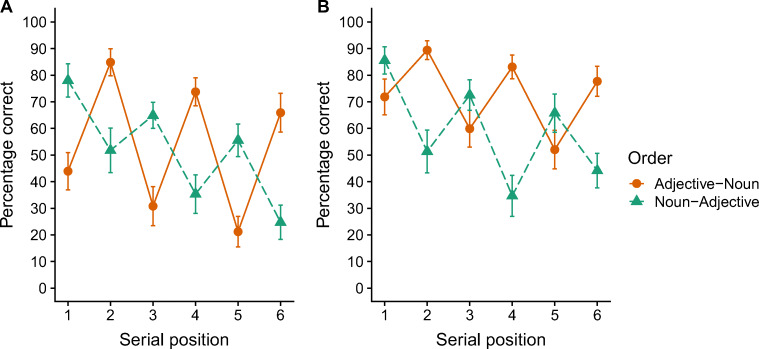
Overall accuracy in Experiment 2 as a function of serial position and order with incongruently inflected adjectives (**a**) and congruently inflected adjectives (**b**). Figures are based on observed data and error bars indicate 95% confidence intervals.

On average, participants recalled 60% (*SD* = 13) of the items in their correct serial position across all conditions. Figure 2 shows the percentage of items correctly recalled as a function of adjective inflection and order (for a serial position plot, see Fig. 4 in the Appendix).

As fixed effects, the model included order, adjective inflection, and version (main effects and all interactions). There were significant main effects for order, β = −0.47325, *SE* = 0.07884, *z* = −6.002, *p* < .001, and adjective inflection, β = −0.74008, *SE* = 0.16290, *z* = −4.543*, p* < .001, while the control variable version did not significantly affect overall recall accuracy, β = 0.07650, *SE* = 0.18787, *z* = 0.407*, p* = .684. Serial recall of lists in adjective–noun order (63%, *SD* = 16) was superior to that of lists in noun–adjective order (56%, *SD* = 13) and accuracy was higher for lists with congruently inflected adjectives (66%, *SD* = 12) than for lists with incongruently inflected adjectives (53%, *SD* = 11).

However, the main effect for order was qualified by an interaction between order and adjective inflection (β = 0.62906, *SE* = 0.16486, *z* = 3.816, *p* < .001). An advantage for adjective–noun order over noun–adjective order was observable only in the condition with congruently inflected adjectives (72%, *SD* = 14 vs. 59%, *SD* = 14, β = 0.788, *SE* = 0.108, *z* = 7.304, *p* < .001), while there was no order difference when adjectives were incongruently inflected (adjective–noun: 53%, *SD* = 11, vs. noun–adjective: 52%, *SD* = 12, β = 0.159, *SE* = 0.120, *z* = 1.323, *p* = .548). Unlike with uninflected adjectives, this interaction reflected an advantage for the most grammatical condition with adjective–noun order and congruently inflected adjectives compared with all other conditions. A likelihood ratio test confirmed that the model including this interaction was significantly better than one without it, χ^2^(2) = 14.492, *p* < .001.^5^ Lastly, none of the other interaction terms in the model was significant (all *p*s > .322).

#### Order errors

The overall proportion of order errors was at 3% (*SD* = 3). Given this low proportion of order errors, no reliable model could be fitted. Descriptively, there were fewer order errors in the condition with congruently inflected adjectives and adjective–noun order (2%, *SD* = 2) than in the condition with congruently inflected adjectives and noun–adjective order (3%, *SD* = 3) and the two conditions with incongruently inflected adjectives (adjective–noun order: 3%, *SD* = 4, noun–adjective order: 5%, *SD* = 4).

As in Experiment 1, we additionally computed conditional probabilities of order errors. Again, these probabilities closely mirrored the descriptive summaries of absolute order errors. The conditional probability of an order error occurring was smallest in the adjective–noun condition with congruent adjective inflection (.039), followed by the noun–adjective condition with congruent adjective inflection (.065) and the adjective–noun condition with incongruently inflected adjectives (.066). The probability of an order error was highest in the condition with noun–adjective order and incongruent adjective inflection (.103). Conditional probabilities of order errors were low overall.

#### Item errors

The overall proportion of item errors was at 37% (*SD* = 12). The logistic mixed-effects model concerning item errors had the same fixed-effects structure as the overall accuracy model. It revealed significant main effects for order, β = 0.41599, *SE* = 0.07032, *z* = 5.916, *p* < .001, and adjective inflection, β = 0.64850, *SE* = 0.15347, *z* = 4.226, *p* < .001. The main effect for the control variable version did not reach significance, β = −0.13169, *SE* = 0.18617, *z* = −0.707, *p* = .479. There were fewer item errors in lists with adjective–noun order (34%, *SD* = 14.5) than in lists with noun–adjective order (40%, *SD* = 11.9). In addition, participants committed fewer item errors in lists with congruently inflected adjectives (32%, *SD* = 11.4) than in lists with incongruently inflected adjectives (43%, *SD* = 9.7). The main effects for order and adjective inflection were, however, qualified by a significant interaction between the two factors, β = −0.63785, *SE* = 0.14962, *z* = −4.263, *p* < .001. The advantage for adjective–noun lists over noun–adjective lists was observable only when the adjectives were congruently inflected (26%, *SD* = 13, vs. 38%, *SD* = 13, β = −0.7349, *SE* = 0.0962, *z* = −7.642, *p* < .001, whereas with incongruently inflected adjectives there was no order difference (adjective–noun: 43%, *SD* = 10 vs. noun–adjective: 43%, *SD* = 10, β = −0.0971, *SE* = 0.1088, *z* = −0.892, *p* = .809). A likelihood ratio test confirmed that the model including this interaction was significantly better than one without it, χ^2^(2) = 17.29, *p* < .001.^6^

### Discussion

We contrasted lists with congruent and incongruent adjective–noun pairs and lists with congruent and incongruent noun–adjective pairs. This was done to exclude the confound of word length between uninflected and inflected adjectives present in Experiment 1, while maintaining the comparison of a condition in which word order was grammatical, but the sequence was nonetheless ungrammatical due to morphosyntactic constraints. As in Experiment 1, there was a significant interaction between order and adjective inflection such that an advantage for the canonical adjective–noun order was observable only with congruently inflected adjectives, but not when the adjective was not congruently inflected with its corresponding noun. Unlike Experiment 1, however, the interaction additionally reflected an advantage for the most grammatical condition—with congruently inflected adjectives and an adjective–noun order—over all other conditions, which in turn did not differ from each other. These findings thus strongly suggest that serial recall benefits from minimal syntactic structure within a list and that syntactic constraints on word order are intertwined with morphosyntactic constraints such as adjective inflection.

## General discussion

In two experiments, we found that in a language that imposes strong morphosyntactic constraints, serial recall performance benefits from a list in which adjectives and nouns are ordered canonically only when the adjective inflection renders the sequence morphosyntactically correct as well. While in English word order alone makes an adjective–noun sequence grammatical (and a noun–adjective sequence ungrammatical), in German, only an adjective preceding a noun with which it is congruently inflected constitutes a grammatical sequence. Consequently, presenting adjectives and nouns in their uninflected form was sufficient for observing an order effect in English (Perham et al., 2009), but not in German (Experiment 1). These findings thus extend Perham et al.’s (2009) findings to a language that is morphologically richer than English and support the idea that minimal syntactic structure within a list helps serial recall, unless morphological constraints undermine the syntactical relation indicated by word order. The fact that the main effects for order and inflection were qualified by the interaction indicates that it is indeed the integration of morphosyntactic word form information and syntactic word order that benefitted serial recall, as emphasized by Schwering and MacDonald (2020). Only when these constraints jointly rendered the sequence grammatical, was there a discernible benefit.

However, the effects on overall accuracy seem to be driven by item errors rather than by order errors. Moreover, unlike overall accuracy and item errors, the analysis on order errors in Experiment 1 did not reveal a significant interaction between order and adjective inflection, but only a small but significant main effect for order and an unexpected interaction between order and version. Notably, order errors were rare in both experiments (4% in Experiment 1 and 3% in Experiment 2, with conditional probabilities of .074 in Experiment 1 and of .068 in Experiment 2). The proportion of order errors was even too small to fit a model to the data in Experiment 2. Thus, the analyses regarding this error type are rather unreliable, and we refrain from interpreting the data pattern. Perham et al. (2009) reported only the data on overall accuracy and refrained from analyzing order and item errors separately since “order errors tend to dominate in this type of setting, hence the analysis of other types of errors was not undertaken” (p. 1288). Given the close methodological approximation of our study, it is plausible that the pattern in overall recall accuracy in their study was also driven by item rather than by order errors. If so, this might be due to the use of an open item set with unique items in each list. Such an item pool demands item memory to a stronger degree than typical materials in verbal serial recall consisting of a limited set of digits, letters, nonwords or words, which are repeated across trials. This is in line with the high proportion of item errors in our Experiments (33% in Experiment 1 and 37% in Experiment 2), which resulted in fewer opportunities for order errors to occur, as order errors were defined as items that were recalled correctly but in an incorrect position. Nonetheless, a straightforward effect of the syntactic order manipulation should have been on order errors. Future studies should thus aim to replicate the current findings with a closed item pool to increase the proportion of order errors.

Item errors included instances in which items were either omitted, substituted by an extra-list item, incorrectly inflected, or substituted by an incorrectly inflected within-list item. A qualitative analysis of the error types can give further insight in specific error patterns in the different conditions—for instance, whether participants tended to regularize adjective inflection when there was a mismatch with the succeeding noun. This would also speak to mechanisms of language comprehension and production as driving forces in recalling these lists.^7^ However, the pattern of item error types only suggests a small degree of regularization. Overall, omissions dominated with both nouns and adjectives, in particular in Experiment 1 (nouns: 79%, adjectives: 72%). In Experiment 2, omissions were still the most frequent type of item error with adjectives (53%). In both experiments, the condition with the highest proportion of inflection errors was the one with noun–adjective order and congruent adjective inflection: Of the adjective item errors, 26% were inflection errors in Experiment 1 and 39% in Experiment 2. However, the inflection errors in these conditions were predominantly cases in which an uninflected adjective was produced instead of an inflected one (Experiment 1: 50%, Experiment 2: 47%). The clearest indication of regularization was observable in the conditions with canonical adjective–noun sequences: Both with uninflected adjectives in Experiment 1 and with incongruently inflected adjectives in Experiment 2, the dominant type of inflection error was an adjustment of adjective inflection to the gender of the noun in the pair (Experiment 1: 83%, Experiment 2: 50%).

So far, we have attributed the difference between the adjective–noun order and the noun–adjective order in the lists with inflected adjectives to an advantage for the well-formed adjective–noun phrases in Positions 1 and 2, 3 and 4, and 5 and 6. However, it is worth noting that there is an alternative explanation, as the inflected adjectives were not only congruent in grammatical gender with their corresponding noun but also incongruent with the other adjacent noun(s) in the list. Thus, the difference could also be driven by the morphosyntactic incompatibility between adjectives and nouns in Positions 2 and 3 and 4 and 5 in the noun–adjective condition. In a noun–adjective list like “Löwe_[masc]_–wässriger_[masc]_–Banane_[fem]_–stürmische_[fem]_–Mantel_[masc]_–besiegter_[masc]_,” these would be the adjective–noun phrases “wässriger_[masc]_ Banane_[fem]_” and “stürmische_[fem]_Mantel_[masc]_.” Even though this grouping was discouraged by the extended interstimulus interval between Positions 2 and 3 and 4 and 5, the gender incongruence might have further hampered such an encoding or maintenance strategy. Given Perham et al.’s (2009) findings in English, in which there was no morphosyntactic incongruence between adjectives and nouns, the order effect for lists with inflected adjectives should also show up when all nouns in a list are of the same grammatical gender, albeit potentially of smaller magnitude.

Even though some open questions remain, our findings indicate that morphosyntactic constraints such as congruency and syntactic constraints such as canonical word order improve serial recall performance when they converge to render sequences within a word list grammatical. In a language that is morphologically richer than English, minimal syntactic structure within a list improves serial recall performance, unless morphological constraints undermine the grammaticality of the canonically ordered sequence. Moreover, participants tend to regularize inflection, when word order suggests that they are parsing an otherwise grammatical sequence.

How can different theories account for these effects? The current results confirm the conclusion from previous findings that the influence of linguistic knowledge goes beyond the individual item level and also acts over multiple items (see also Allen et al., 2018; Jefferies et al., 2004; T. Jones & Farrell, 2018; Perham et al., 2009). Consequently, T. Jones and Farrell (2018) stated that “contemporary positional models of serial ordering in short-term memory . . . offer no obvious mechanism to account for such sequential effects. To account for the effects shown here, such models must be extended to include mechanisms of support from long-term representations acting over multiple items” (p. 119f.). As described above, a core mechanism with which the sentence superiority effect has been explained is chunking (Allen et al., 2018; Baddeley et al., 2009). However, chunking mechanisms as currently described (e.g., Oberauer et al., 2018) or as implemented in computational models (e.g., Burgess & Hitch, 2005), rely on known combinations of specific items as observable in collocation frequencies. In both experiments, we selected materials such that the specific word combinations were semantically implausible. Consequently, they should not have been frequently encountered in combination in previous experience.

In line with the study by Perham et al. (2009; see also T. Jones & Farrell, 2018), our findings thus imply a more abstract ordering constraint that operates on word classes rather than on specific lexical entries, as is implied in language-based accounts of verbal short-term memory (e.g., Acheson & MacDonald, 2009; MacDonald, 2016; Majerus, 2013; Perham et al., 2009). Moreover, the finding that incompatible adjective inflection impeded the order effect suggests that such constraints do not operate solely based on word classes, but on word classes that are morphosyntactically specified.

With respect to the distinction between “limited emergent” and “rich emergent” models, the current findings are in line with the account put forward by Schwering and MacDonald (2020). Though not designed to test their assumptions, the current experiments provide a case in which their claim that, due to the highly intertwined character of language processing, item knowledge is hard to be “separated from order knowledge if the source of the order benefit is derived from the association with the individual words” (Schwering & MacDonald, 2020, p. 6). This is indeed the case in our experiments, in which the combination of a syntactically based order manipulation (adjective–noun vs. noun–adjective) and a morphosyntactically based item manipulation (adjective inflection) benefitted performance in a serial recall task—feat that neither manipulation alone accomplished. In particular, the order manipulation had no effect on sequences in which the adjectives were either uninflected or inflected incongruently with both adjacent nouns. Although the current findings are clearly in line with Schwering and MacDonald’s (2020) account, they do not contradict other types of language-based models that still assume a distinction between item and order memory. Majerus (2013), for instance, assumes a distinct mechanism for maintaining order in lists with arbitrary or unfamiliar order. From this perspective, item memory as well as order memory within an adjective–noun sequence with congruently inflected adjectives may have benefitted from language experience. In contrast, the ordering within noun–adjective sequences and adjective–noun sequences with either uninflected or incongruently inflected adjectives would have invoked a separate ordering component. A separate ordering mechanism might also have accomplished the ordering between the three pairs in all conditions.

Our study was conducted in a language in which grammaticality depends to a high degree on inflection. Whether incompatible inflection impedes order effects in English as well cannot yet be concluded, because in previous studies, either correctly inflected verb forms or uninflected but compatible adjective forms were used (Allen et al., 2018; T. Jones & Farrell, 2018; Perham et al., 2009). It therefore remains to be investigated whether beneficial effects of familiar word order and/or of correct inflection vary between languages depending on how flexible their word order is and how strict the difference between correct and incorrect word order is. One implication of language-based theories is that the relative importance of word order and inflection for verbal short-term memory is directly bound to its importance for grammaticality in the given language. These considerations demonstrate the relevance of investigating the influence of linguistic long-term knowledge on verbal short-term memory in languages other than English.

## Appendix

**Table 1:** Materials used in Experiment 1: Lists with noun–adjective pairs and uninflected adjectives (Version A), including English translations (in parentheses) and grammatical gender of the nouns [in brackets]

List	Item 1	Item 2	Item 3	Item 4	Item 5	Item 6
1	Fenster_[n]_ (window)	juckend (itchy)	Pinguin_[m]_ (penguin)	sonnig (sunny)	Mandel_[f]_ (almond)	neblig (misty)
2	Lachs_[m]_ (salmon)	frech (cheeky)	Lampe_[f]_ (lamp)	schleimig (slimy)	Zeh_[n]_ (toe)	heiter (cheerful)
3	Karpfen_[m]_ (carp)	müde (tired)	Jacke_[f]_ (jacket)	blass (pale)	Essig_[m]_ (vinegar)	hohl (hollow)
4	Gepard_[m]_ (cheetah)	beschämt (ashamed)	Maschine_[f]_ (engine)	kahl (bald)	Rücken_[m]_ (back)	mies (miserable)
5	Hai_[m]_ (shark)	staubig (dusty)	Amsel_[f]_ (blackbird)	krumm (bent)	Wein_[m]_ (wine)	brutal (brutal)
6	Strauß_[m]_ (bouquet)	nervös (nervous)	Blume_[f]_ (flower)	luftig (airy)	Schlips_[f]_ (tie)	salzig (salty)
7	Schaf_[n]_ (sheep)	lustig (funny)	Hand_[f]_ (hand)	ängstlich (afraid)	Hemd_[n]_ (shirt)	knusprig (crispy)
8	Bein_[n]_ (leg)	ruhig (quiet)	Knoblauch_[m]_ (garlic)	eckig (square)	Gelenk_[n]_ (joint)	weich (soft)
9	Vitrine_[f]_ (showcase)	trocken (dry)	Spiegel_[m]_ (mirror)	albern (foolish)	Achse_[f]_ (axle)	bitter (bitter)
10	Laken_[n]_ (sheets)	holzig (woody)	Rock_[m]_ (skirt)	aktiv (active)	Tuch_[n]_ (scarf)	mehlig (floury)
11	Kröte_[f]_ (toad)	trotzig (defiant)	Kleid_[n]_ (dress)	schüchtern (shy)	Balkon_[m]_ (balcony)	gezielt (aimed)
12	Adler_[m]_ (eagle)	schick (fancy)	Rose_[f]_ (rose)	flach (flat)	Motor_[m]_ (motor)	bewölkt (cloudy)

**Table 2:** Materials used in Experiment 1: Lists with adjective–noun pairs and uninflected adjectives (sVersion A), including English translations (in parentheses) and grammatical gender of the nouns [in brackets]

List	Item 1	Item 2	Item 3	Item 4	Item 5	Item 6
1	wässrig (watery)	Löwe_[m]_ (lion)	stürmisch (stormy)	Banane_[f]_ (banana)	besiegt (defeated)	Mantel_[m]_ (coat)
2	mutig (brave)	Dach_[n]_ (roof)	unschuldig (innocent)	Korridor_[m]_ (hallway)	mollig (chubby)	Lilie_[f]_ (lily)
3	dankbar (grateful)	Bier_[n]_ (beer)	geizig (stingy)	Schulter_[f]_ (shoulder)	tief (deep)	Elefant_[m]_ (elephant)
4	fröhlich (jolly)	Hering_[m]_ (herring)	dumm (silly)	Regal_[n]_ (shelf)	locker (loose)	Tisch_[m]_ (table)
5	witzig (funny)	Delfin_[m]_ (dolphin)	niedrig (low)	Kuh_[f]_ (cow)	rot (red)	Tequila_[m]_ (tequila)
6	loyal (faithful)	Samt_[m]_ (velvet)	breiig (mushy)	Tulpe_[f]_ (tulip)	leicht (easy)	Gürtel_[m]_ (belt)
7	gemein (mean)	Reh_[n]_ (deer)	schmal (slim)	Batterie_[f]_ (battery)	schwach (weak)	Zucker_[m]_ (sugar)
8	ehrlich (honest)	Silikon_[n]_ (silicone)	robust (robust)	Butter_[f]_ (butter)	rutschig (slippery)	Pferd_[n]_ (horse)
9	froh (glad)	Hals_[m]_ (neck)	töricht (stupid)	Ziege_[f]_ (goat)	tapsig (clumsy)	Kopf_[m]_ (head)
10	glücklich (happy)	Schuh_[m]_ (shoe)	neidisch (jealous)	Himbeere_[f]_ (raspberry)	windig (breezy)	Schwein_[n]_ (pig)
11	verrückt (crazy)	Wodka_[m]_ (vodka)	fettig (greasy)	Herz_[n]_ (heart)	straff (tense)	Schnaps_[m]_ (liquor)
12	gestreift (striped)	Soße_[f]_ (sauce)	eifrig (eager)	Falke_[m]_ (falcon)	lockig (curly)	Bett_[n]_ (bed)

**Table 3:** Materials used in Experiment 1: Lists with noun–adjective pairs and inflected adjectives (Version A), including English translations (in parentheses) and grammatical gender [in brackets]

List	Item 1	Item 2	Item 3	Item 4	Item 5	Item 6
1	Fenster_[n]_ (window)	juckendes_[n]_ (itchy)	Pinguin_[m]_ (penguin)	sonniger_[m]_ (sunny)	Mandel_[f]_ (almond)	neblige_[f]_ (misty)
2	Lachs_[m]_ (salmon)	frecher_[m]_ (cheeky)	Lampe_[f]_ (lamp)	schleimige_[f]_ (slimy)	Zeh_[m]_ (toe)	heiterer_[m]_ (cheerful)
3	Karpfen_[m]_ (carp)	müder_[m]_ (tired)	Jacke_[f]_ (jacket)	blasse_[f]_ (pale)	Essig_[m]_ (vinegar)	hohler_[m]_ (hollow)
4	Gepard_[m]_ (cheetah)	beschämter_[m]_ (ashamed)	Maschine_[f]_ (engine)	kahle_[f]_ (bald)	Rücken_[m]_ (back)	mieser_[m]_ (miserable)
5	Hai_[m]_ (shark)	staubiger_[m]_ (dusty)	Amsel_[f]_ (blackbird)	krumme_[f]_ (bent)	Wein_[m]_ (wine)	brutaler_[m]_ (brutal)
6	Strauß_[m]_ (bouquet)	nervöser_[m]_ (nervous)	Blume_[f]_ (flower)	luftige_[f]_ (airy)	Schlips_[f]_ (tie)	salziger_[f]_ (salty)
7	Schaf_[n]_ (sheep)	lustiges_[n]_ (funny)	Hand_[f]_ (hand)	ängstliche_[f]_ (afraid)	Hemd_[n]_ (shirt)	knuspriges_[n]_ (crispy)
8	Bein_[n]_ (leg)	ruhiges_[n]_ (quiet)	Knoblauch_[m]_ (garlic)	eckiger_[m]_ (square)	Gelenk_[n]_ (joint)	weiches_[n]_ (soft)
9	Vitrine_[f]_ (showcase)	trockene_[f]_ (dry)	Spiegel_[m]_ (mirror)	alberner_[m]_ (foolish)	Achse_[f]_ (axle)	bittere_[f]_ (bitter)
10	Laken_[n]_ (sheets)	holziges_[n]_ (woody)	Rock_[m]_ (skirt)	aktiver_[m]_ (active)	Tuch_[n]_ (scarf)	mehliges_[n]_ (floury)
11	Kröte_[f]_ (toad)	trotzige_[f]_ (defiant)	Kleid_[n]_ (dress)	schüchternes_[n]_ (shy)	Balkon_[m]_ (balcony)	gezielter_[m]_ (aimed)
12	Adler_[m]_ (eagle)	schicker_[m]_ (fancy)	Rose_[f]_ (rose)	flache_[f]_ (flat)	Motor_[m]_ (motor)	bewölkter_[m]_ (cloudy)

**Table 4:** Materials used in Experiment 1: Lists with adjective–noun pairs and uninflected adjectives (Version A), including English translations (in parentheses) and grammatical gender [in brackets]

List	Item 1	Item 2	Item 3	Item 4	Item 5	Item 6
1	wässriger_[m]_ (watery)	Löwe_[m]_ (lion)	stürmische_[f]_ (stormy)	Banane_[f]_ (banana)	besiegter_[m]_ (defeated)	Mantel_[m]_ (coat)
2	mutiges_[n]_ (brave)	Dach_[n]_ (roof)	unschuldiger_[m]_ (innocent)	Korridor_[m]_ (hallway)	mollige_[f]_ (chubby)	Lilie_[f]_ (lily)
3	dankbares_[n]_ (grateful)	Bier_[n]_ (beer)	geizige_[f]_ (stingy)	Schulter_[f]_ (shoulder)	tiefer_[m]_ (deep)	Elefant_[m]_ (elephant)
4	fröhlicher_[m]_ (jolly)	Hering_[m]_ (herring)	dummes_[n]_ (silly)	Regal_[n]_ (shelf)	lockerer_[m]_ (loose)	Tisch_[m]_ (table)
5	witziger_[m]_ (funny)	Delfin_[m]_ (dolphin)	niedrige_[f]_ (low)	Kuh_[f]_ (cow)	roter_[m]_ (red)	Tequila_[m]_ (tequila)
6	loyaler_[m]_ (faithful)	Samt_[m]_ (velvet)	breiige_[f]_ (mushy)	Tulpe_[f]_ (tulip)	leichter_[m]_ (easy)	Gürtel_[m]_ (belt)
7	gemeines_[n]_ (mean)	Reh_[n]_ (deer)	schmale_[f]_ (slim)	Batterie_[f]_ (battery)	schwacher_[m]_ (weak)	Zucker_[m]_ (sugar)
8	ehrliches_[n]_ (honest)	Silikon_[n]_ (silicone)	robuste_[f]_ (robust)	Butter_[f]_ (butter)	rutschiges_[n]_ (slippery)	Pferd_[n]_ (horse)
9	froher_[m]_ (glad)	Hals_[m]_ (neck)	törichte_[f]_ (stupid)	Ziege_[f]_ (goat)	tapsiger_[m]_ (clumsy)	Kopf_[m]_ (head)
10	glücklicher_[m]_ (happy)	Schuh_[m]_ (shoe)	neidische_[f]_ (jealous)	Himbeere_[f]_ (raspberry)	windiges_[n]_ (breezy)	Schwein_[n]_ (pig)
11	verrückter_[m]_ (crazy)	Wodka_[m]_ (vodka)	fettiges_[n]_ (greasy)	Herz_[n]_ (heart)	straffer_[m]_ (tense)	Schnaps_[m]_ (liquor)
12	gestreifte_[f]_ (striped)	Soße_[f]_ (sauce)	eifriger_[m]_ (eager)	Falke_[m]_ (falcon)	lockiges_[n]_ (curly)	Bett_[n]_ (bed)

**Table 5:** Materials used in Experiment 2: Lists with noun–adjective pairs and congruently inflected adjectives (Version A), including English translations (in parentheses) and grammatical gender [in brackets]

List	Item 1	Item 2	Item 3	Item 4	Item 5	Item 6
1	Löwe_[m]_ (lion)	wässriger_[m]_ (watery)	Banane_[f]_ (banana)	stürmische_[f]_ (stormy)	Mantel_[m]_ (coat)	besiegter_[m]_ (defeated)
2	Dach_[n]_ (roof)	mutiges_[n]_ (brave)	Korridor_[m]_ (corridor)	unschuldiger_[m]_ (innocent)	Lilie_[f]_ (lily)	mollige_[f]_ (chubby)
3	Bier_[n]_ (beer)	dankbares_[n]_ (grateful)	Schulter_[f]_ (shoulder)	geizige_[f]_ (stingy)	Elefant_[m]_ (elephant)	tiefer_[m]_ (deep)
4	Hering_[m]_ (herring)	fröhlicher_[m]_ (happy)	Regal_[n]_ (shelf)	dummes_[n]_ (silly)	Tisch_[m]_ (table)	lockerer_[m]_ (loose)
5	Delfin_[m]_ (dolphin)	witziger_[m]_ (funny)	Kuh_[f]_ (cow)	niedrige_[f]_ (low)	Tequila_[m]_ (tequila)	roter_[m]_ (red)
6	Stoff_[m]_ (fabric)	loyaler_[m]_ (faithful)	Tulpe_[f]_ (tulip)	breiige_[f]_ (mushy)	Turban_[m]_ (turban)	leichter_[m]_ (easy)
7	Reh_[n]_ (deer)	gemeines_[n]_ (mean)	Batterie_[f]_ (battery)	schmale_[f]_ (slim)	Zucker_[m]_ (sugar)	schwacher_[m]_ (weak)
8	Silikon_[n]_ (silicone)	ehrliches_[n]_ (honest)	Butter_[f]_ (butter)	robuste_[f]_ (robust)	Pferd_[n]_ (horse)	rutschiges_[n]_ (slippery)
9	Hals_[m]_ (neck)	froher_[m]_ (glad)	Ziege_[f]_ (goat)	törichte_[f]_ (stupid)	Kopf_[m]_ (head)	tapsiger_[m]_ (clumsy)
10	Schuh_[m]_ (shoe)	glücklicher_[m]_ (happy)	Himbeere_[f]_ (raspberry)	neidische_[f]_ (jealous)	Schwein_[n]_ (pig)	windiges_[n]_ (breezy)
11	Wodka_[m]_ (vodka)	verrückter_[m]_ (crazy)	Herz_[n]_ (heart)	fettiges_[n]_ (greasy)	Schnaps_[m]_ (liquor)	straffer_[m]_ (tense)
12	Soße_[f]_ (sauce)	gestreifte_[f]_ (striped)	Falke_[m]_ (hawk)	eifriger_[m]_ (eager)	Bett_[n]_ (bed)	lockiges_[n]_ (curly)

**Table 6:** Materials used in Experiment 2: Lists with adjective–noun pairs and congruently inflected adjectives (Version A), including English translations (in parentheses) and grammatical gender[in brackets]

List	Item 1	Item 2	Item 3	Item 4	Item 5	Item 6
1	juckendes_[n]_ (itchy)	Parkett_[n]_ (parquet)	sonniger_[m]_ (sunny)	Pinguin_[m]_ (penguin)	neblige_[f]_ (misty)	Mandel_[f]_ (almond)
2	frecher_[m]_ (cheeky)	Lachs_[m]_ (salmon)	schleimige_[f]_ (slimy)	Lampe_[f]_ (lamp)	heiterer_[m]_ (cheerful)	Zeh_[m]_ (toe)
3	müder_[m]_ (tired)	Orca_[m]_ (orca)	blasse_[f]_ (pale)	Jacke_[f]_ (jacket)	hohler_[m]_ (hollow)	Essig_[m]_ (vinegar)
4	beschämter_[m]_ (ashamed)	Gepard_[m]_ (cheetah)	kahle_[f]_ (bare)	Maschine_[f]_ (machine)	mieser_[m]_ (miserable)	Rücken_[m]_ (back)
5	staubiger_[m]_ (dusty)	Hai_[m]_ (shark)	krumme_[f]_ (bent)	Amsel_[f]_ (blackbird)	brutaler_[m]_ (brutal)	Wein_[m]_ (wine)
6	nervöser_[m]_ (nervous)	Strauß_[m]_ (bouquet)	luftige_[f]_ (airy)	Blume_[f]_ (flower)	salziger_[m]_ (salty)	Schlips_[m]_ (tie)
7	lustiges_[n]_ (funny)	Schaf_[n]_ (sheep)	ängstliche_[f]_ (afraid)	Hand_[f]_ (hand)	knuspriges_[n]_ (crispy)	Hemd_[n]_ (shirt)
8	ruhiges_[n]_ (quiet)	Bein_[n]_ (leg)	eckiger_[m]_ (square)	Knoblauch_[m]_ (garlic)	weiches_[n]_ (soft)	Gelenk_[n]_ (joint)
9	trockene_[f]_ (dry)	Vitrine_[f]_ (showcase)	alberner_[m]_ (foolish)	Spiegel_[m]_ (mirror)	bittere_[f]_ (bitter)	Achse_[f]_ (axle)
10	holziges_[n]_ (woody)	Sakko_[n]_ (jacket)	aktiver_[m]_ (active)	Rock_[m]_ (skirt)	mehliges_[n]_ (floury)	Tuch_[n]_ (scarf)
11	trotzige_[f]_ (defiant)	Kröte_[f]_ (toad)	schüchternes_[n]_ (shy)	Kleid_[n]_ (dress)	gezielter_[m]_ (aimed)	Balkon_[m]_ (balcony)
12	schicker_[m]_ (fancy)	Bussard_[m]_ (buzzard)	flache_[f]_ (flat)	Rose_[f]_ (rose)	bewölkter_[m]_ (cloudy)	Motor_[m]_ (motor)

**Table 7:** Materials used in Experiment 2: Lists with noun–adjective pairs and incongruently inflected adjectives (Version A), including English translations (in parentheses) and grammatical gender [in brackets]

List	Item 1	Item 2	Item 3	Item 4	Item 5	Item 6
1	Löwe_[m]_ (lion)	wässriges_[n]_ (watery)	Banane_[f]_ (banana)	stürmisches_[n]_ (stormy)	Mantel_[m]_ (coat)	besiegte_[f]_ (defeated)
2	Dach_[n]_ (roof)	mutige_[f]_ (brave)	Korridor_[m]_ (corridor)	unschuldiges_[n]_ (innocent)	Lilie_[f]_ (lily)	molliges_[n]_ (chubby)
3	Bier_[n]_ (beer)	dankbarer_[m]_ (grateful)	Schulter_[f]_ (shoulder)	geiziges_[n]_ (stingy)	Elefant_[m]_ (elephant)	tiefes_[n]_ (deep)
4	Hering_[m]_ (herring)	fröhliche_[f]_ (happy)	Regal_[n]_ (shelf)	dumme_[f]_ (silly)	Tisch_[m]_ (table)	lockeres_[n]_ (loose)
5	Delfin_[m]_ (dolphin)	witziges_[n]_ (funny)	Kuh_[f]_ (cow)	niedriges_[n]_ (low)	Tequila_[m]_ (tequila)	rote_[f]_ (red)
6	Stoff_[m]_ (fabric)	loyales_[n]_ (faithful)	Tulpe_[f]_ (tulip)	breiiges_[n]_ (mushy)	Turban_[m]_ (turban)	leichte_[f]_ (easy)
7	Reh_[n]_ (deer)	gemeiner_[m]_ (mean)	Batterie_[f]_ (battery)	schmales_[n]_ (slim)	Zucker_[m]_ (sugar)	schwaches_[n]_ (weak)
8	Silikon_[n]_ (silicone)	ehrlicher_[m]_ (honest)	Butter_[f]_ (butter)	robuster_[m]_ (robust)	Pferd_[n]_ (horse)	rutschige_[f]_ (slippery)
9	Hals_[m]_ (neck)	frohes_[n]_ (glad)	Ziege_[f]_ (goat)	törichtes_[n]_ (stupid)	Kopf_[m]_ (head)	tapsige_[f]_ (clumsy)
10	Schuh_[m]_ (shoe)	glückliches_[n]_ (happy)	Himbeere_[f]_ (raspberry)	neidischer_[m]_ (jealous)	Schwein_[n]_ (pig)	windiger_[m]_ (breezy)
11	Wodka_[m]_ (vodka)	verrückte_[f]_ (crazy)	Herz_[n]_ (heart)	fettige_[f]_ (greasy)	Schnaps_[m]_ (liquor)	straffes_[n]_ (tense)
12	Soße_[f]_ (sauce)	gestreiftes_[n]_ (striped)	Falke_[m]_ (hawk)	eifrige_[f]_ (eager)	Bett_[n]_ (bed)	lockige_[f]_ (curly)

**Table 8:** Materials used in Experiment 2: Lists with adjective–noun pairs and incongruently inflected adjectives (Version A), including English translations (in parentheses) and grammatical gender [in brackets]

List	Item 1	Item 2	Item 3	Item 4	Item 5	Item 6
1	juckender_[m]_ (itchy)	Parkett_[n]_ (parquet)	sonnige_[f]_ (sunny)	Pinguin_[m]_ (penguin)	nebliges_[n]_ (misty)	Mandel_[f]_ (almond)
2	freche_[f]_ (cheeky)	Lachs_[m]_ (salmon)	schleimiges_[n]_ (slimy)	Lampe_[f]_ (lamp)	heiteres_[n]_ (cheerful)	Zeh_[m]_ (toe)
3	müde_[f]_ (tired)	Orca_[m]_ (orca)	blasses_[n]_ (pale)	Jacke_[f]_ (jacket)	hohles_[n]_ (hollow)	Essig_[m]_ (vinegar)
4	beschämte_[f]_ (ashamed)	Gepard_[m]_ (cheetah)	kahles_[n]_ (bare)	Maschine_[f]_ (machine)	mieses_[n]_ (miserable)	Rücken_[m]_ (back)
5	staubige_[f]_ (dusty)	Hai_[m]_ (shark)	krummes_[n]_ (bent)	Amsel_[f]_ (blackbird)	brutales_[n]_ (brutal)	Wein_[m]_ (wine)
6	nervöse_[f]_ (nervous)	Strauß_[m]_ (bouquet)	luftiges_[n]_ (airy)	Blume_[f]_ (flower)	salziges_[n]_ (salty)	Schlips_[m]_ (tie)
7	lustige_[f]_ (funny)	Schaf_[n]_ (sheep)	ängstlicher_[m]_ (afraid)	Hand_[f]_ (hand)	knuspriger_[m]_ (crispy)	Hemd_[n]_ (shirt)
8	ruhiger_[m]_ (quiet)	Bein_[n]_ (leg)	eckige_[f]_ (square)	Knoblauch_[m]_ (garlic)	weiche_[f]_ (soft)	Gelenk_[n]_ (joint)
9	trockener_[m]_ (dry)	Vitrine_[f]_ (showcase)	albernes_[n]_ (foolish)	Spiegel_[m]_ (mirror)	bitteres_[n]_ (bitter)	Achse_[f]_ (axle)
10	holziger_[m]_ (woody)	Sakko_[n]_ (jacket)	aktive_[f]_ (active)	Rock_[m]_ (skirt)	mehlige_[f]_ (floury)	Tuch_[n]_ (scarf)
11	trotziger_[m]_ (defiant)	Kröte_[f]_ (toad)	schüchterner_[m]_ (shy)	Kleid_[n]_ (dress)	gezielte_[f]_ (aimed)	Balkon_[m]_ (balcony)
12	schicke_[f]_ (fancy)	Bussard_[m]_ (buzzard)	flaches_[n]_ (flat)	Rose_[f]_ (rose)	bewölktes_[n]_ (cloudy)	Motor_[m]_ (motor)

## Footnotes

1 Note that it was necessary to additionally drop the three-way interaction term in the reduced model that was compared with the full model here (hence, 2 degrees of freedom). However, additional analyses showed that the three-way interaction did not significantly improve the full model’s performance, χ^2^(1) = 0.1346, *p* = .7137, and the two-way interaction order × adjective inflection was still significant when comparing its inclusion versus exclusion within such a full model lacking the three-way interaction, χ^2^(1) = 25.842, *p* < .001.

2 Note that as for the overall accuracy model, it was necessary to additionally drop the three-way interaction term in the reduced model that was compared with the full model here. However, additional analyses showed that the three-way interaction did not significantly improve the full model’s performance, χ^2^(1) = 0.0313, *p* = .859, and the two-way interaction order x list version was still significant when comparing its inclusion versus exclusion within such a full model lacking the three-way interaction, χ^2^(1) = 9.8999, *p* = .002.

3 We thank Steve Schwering and Maryellen MacDonald for this suggestion.

4 Note that as in previous models, it was necessary to additionally drop the three-way interaction term in the reduced model that was compared with the full model here. However, additional analyses showed that the three-way interaction did not significantly improve the full model’s performance, χ^2^(1) = 0.4146, *p*= .5196), and the two-way Order × Adjective Inflection interaction was still significant when comparing its inclusion versus exclusion within such a full model lacking the three-way interaction, χ^2^(1) = 26.342, *p* < .001.

5 Note that it was necessary to additionally drop the three-way interaction term in the reduced model that was compared with the full model here. However, additional analyses showed that the three-way interaction did not significantly improve the full model’s performance, χ^2^(1) = 0.9756, *p* = .323, and the two-way Order × Adjective Inflection interaction was still significant when comparing its inclusion versus exclusion within such a full model lacking the three-way interaction, χ^2^(1) = 13.516, *p* < .001.

6 Note that it was necessary to additionally drop the three-way interaction term in the reduced model that was compared with the full model here. However, additional analyses showed that the three-way interaction did not significantly improve the full model’s performance, χ^2^(1) = 0.8196, *p* = .365, and the two-way Order × Adjective Inflection interaction was still significant when comparing its inclusion versus exclusion within such a full model lacking the three-way interaction, χ^2^(1) = 16.471, *p* < .001.

7 We thank an anonymous reviewer for suggesting a qualitative analysis of error types. We have included detailed tables reporting the descriptive item error analyses in the OSF project.
